# A literature review on the role of *Culicoides* in the transmission of avian blood parasites in Europe

**DOI:** 10.1186/s13071-025-06957-y

**Published:** 2025-08-03

**Authors:** Carolina Romeiro Fernandes Chagas, Rasa Bernotienė, Aneliya Bobeva, Dovilė Bukauskaitė, Martina Ferraguti, Rafael Gutiérrez-Lopez, Margarita Kazak, Bruno Mathieu, Kristina Valavičiūte-Pocienė, Diego Santiago-Alarcon, Milena Svobodová, Jesús Veiga, Jan Votýpka, Rita Žiegytė, Josué Martínez-de la Puente

**Affiliations:** 1 State Scientific Research Institute Nature Research Centre, Vilnius, Lithuania; 2 Institute of Biodiversity and Ecosystem Research, Sofia, Bulgaria; 3 Estación Biológica de Doñana (EBD, CSIC), Seville, Spain; 4 Centro de Investigación Biomédica en Red de Epidemiología y Salud Pública (CIBERESP), Madrid, Spain; 5 National Center of Microbiology, National Institute of Health Carlos III, Madrid, Spain; 6 Consorcio de Investigación Biomédica en Red de Enfermedades Infeciosas (CIBERINFEC), Madrid, Spain; 7 Institute of Bacteriology and Parasitology, UR 3073 PHAVI, Medical Faculty, University of Strasbourg, Strasbourg, France 67000; 8 Department of Integrative Biology, University of South Florida, Tampa, USA; 9 Department of Parasitology, Faculty of Science, Charles University, Prague, Czechia

**Keywords:** Avian malaria, Birds, Blood parasites, *Culicoides*, *Haemoproteus*, Vectors, *Trypanosoma*

## Abstract

*Culicoides* (Diptera: Ceratopogonidae) biting midges are a diverse group of insect vectors that transmit pathogens affecting humans, livestock, and wild animals. Among them, Oropouche virus, African Horse sickness virus, and bluetongue virus are the most notable pathogens. However, comparatively little is known about which *Culicoides* species serve as vectors of wildlife parasites affecting wild birds globally, including the malaria-like parasite of the genus *Haemoproteus* (Haemosporida: Haemoproteidae) and kinetoplastid *Trypanosoma* (Trypanosomatida: Trypanosomatidae). Beyond the direct impact of their bites, infections by these parasites negatively affect wild birds from early developmental stages, significantly influencing their ecology and evolution. Here, we present a comprehensive review of the role of *Culicoides* species in the transmission of these two genera of avian parasites in Europe: *Haemoproteus* and *Trypanosoma*. We identify key information and methods used to study *Culicoides*–bird–parasite interactions, from insect sampling to vector competence assessment. Additionally, we highlight key knowledge gaps and propose future research directions in this area.

**Graphical Abstract:**

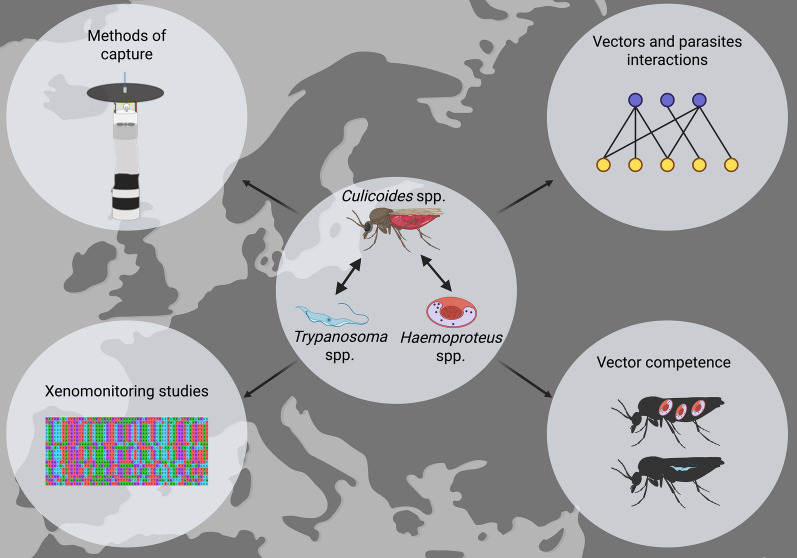

**Supplementary Information:**

The online version contains supplementary material available at 10.1186/s13071-025-06957-y.

## Background

Biting midges of the genus *Culicoides* (Diptera: Ceratopogonidae) are small flying hematophagous insects, typically measuring 1–3 mm in length. The genus is diverse, with 1347 species described worldwide [1], which are broadly distributed across a variety of habitats on the planet, with some exceptions, such as remote islands and Antarctica. Similarly to other nematoceran hematophagous Diptera, only female biting midges take blood meals. Interestingly, some *Culicoides* species, such as *Culicoides anopheles* Edwards, 1922, can feed on blood contained in the abdomen of engorged mosquitoes, although the possibility that this species also feeds on the hemolymph of the mosquitoes has been discussed [2]. Despite these observations, most *Culicoides* species obtain their blood meals from vertebrates, mainly mammals and birds [3–9], although some feed on reptiles [10] and amphibians [11].

The blood-feeding patterns of biting midges differ among *Culicoides* subgenera. Some predominantly feed on mammals (e.g., *Avaritia*,* Monoculicoides*, *Culicoides*, *Silvaticulicoides*), while others include a higher proportion of avian blood meals in their diet (e.g., *Oecacta*, *Beltranmyia*, *Wirthomyia*) [6]. These feeding preferences are likely influenced by variations in the morphology of sensory organs, particularly the maxillary palp, as well as the distribution and density of sensilla on the antennae [12, 13].

In addition to the skin injuries caused during blood feeding, mainly studied in livestock [14], *Culicoides* play a crucial role as vectors of medical- and veterinary-relevant pathogens [15, 16]. The most relevant *Culicoides*-borne viruses is the Oropouche virus, which has recently caused outbreaks in Latin America [17, 18]. Additionally, *Culicoides* transmit pathogens responsible for livestock diseases, such as African horse sickness, bluetongue, and epizootic haemorrhagic disease [19, 20].

Although comparatively less studied, *Culicoides* are also vectors of parasites affecting wildlife, including the avian malaria-like *Haemoproteus* (Haemosporida: Haemosporidae) as well as avian *Trypanosoma* (Trypanosomatida: Trypanosomatidae) [21–32]. The abundance and biting rate of *Culicoides* may influence the transmission risk of blood parasites to birds, even from the early stages of chick development [33]. In addition to the nuisance caused by *Culicoides* bites [34], infection by *Culicoides*-borne parasites, which is the case for *Haemoproteus*, can negatively impact the general health status, reproductive success, and survival probability of wild birds [35–37]. Some of these parasites, such as *Haemoproteus*, can be also virulent for blood-sucking insects and can even cause the mortality of vectors [38, 39]. On the other hand, *Trypanosoma* infections are considered mainly harmless to their hosts and vectors [40], but the long-term effects of such infections remain unexplored.

Here, we review the current knowledge on *Culicoides* as avian blood feeders and vectors of two major avian parasite genera, *Haemoproteus* and *Trypanosoma.* This article focuses on the various methods used to identify and investigate bird–*Culicoides*–parasite interactions in Europe, a region where these interactions have been studied in greater depth.

## Capture of bird-biting *Culicoides*

Different approaches have been used to capture *Culicoides* midges and investigate their role as vectors of avian blood parasites. These methods include (i) direct exposure of birds to *Culicoides* in the field where these insects are abundant [29, 41–50]; (ii) collecting *Culicoides* directly from or close to birds’ nests [32, 51–56]; (iii) using ultraviolet (UV) light traps to collect *Culicoides* at night [3, 7, 8, 24, 25, 31, 32, 57–60]; and (iv) Centers for Disease Control and Prevention (CDC) traps baited with birds [61] or without any bait [62].

Direct exposure experiments have shown that *Culicoides impunctatus* Goetghebuer, 1920, can feed on various passerine hosts (12 species belonging to seven different families) and even from different orders, such as owls [43, 46, 48–50, 63, 64].

Various methods have also been developed to capture *Culicoides* in avian nests. One method involves using Petri dishes coated with body gel oil to trap *Culicoides* females inside nest boxes occupied by passerines [32, 54]. Another approach employs sticky paper traps for the same purpose [56]. Both techniques have successfully captured *Culicoides* females of different physiological status, including nulliparous, gravid, parous, and blood-engorged individuals, as well as, occasionally, some *Culicoides* males, which can aid in species identification [65]. These methods have provided valuable insights into the biting midge species that attack birds during their reproductive period across different bird species and geographical regions [51]. For instance, in blue tit (*Cyanistes caeruleus* Linnaeus, 1758) nests from central Spain, seven *Culicoides* species were identified, including *Culicoides simulator* Edwards, 1939*, Culicoides kibunensis* Tokunaga, 1937, *Culicoides festivipennis* Kieffer, 1914, *Culicoides segnis* Campbell & Pelham-Clinton, 1960, *Culicoides truncorum* Edwards, 1939, *Culicoides pictipennis* Staeger, 1839, and *Culicoides circumscriptus* Kieffer, 1918 [65]. Similarly, in Kaliningrad Oblast, Russia, the same methodology captured *C. kibunensis*, *C. pictipennis*, and *C. segnis* [32].

*Culicoides* likely use various cues to locate avian hosts within their nests, including temperature [66] and metabolic gases such as CO_2_ [67]. Biting midges captured in avian nests provide valuable material for molecular investigation on feeding preferences based on the sex of the nestlings bitten, as well as identifying which individuals the insects are more attracted to [68]. Additionally, they can be used to molecularly detect the presence and identity of avian parasites in parous and recently engorged *Culicoides* females [7, 8, 55, 69].

Additional insights into *Culicoides* ecology and their role as avian parasite vectors have been gained through captures using methods such as CDC, BG-Pro (Biogents, Germany), and Onderstepoort traps, with or without UV light [24, 25, 30, 31, 55, 57–60, 62, 70, 71]. In addition to these methods, which are commonly used to collect *Culicoides* in the field, BG-Sentinel (Biogents, Germany) traps baited with CO_2_ have also proven effective for sampling *Culicoides*, including ornithophilic species. For instance, the recently described species *Culicoides grandifovea* González, Magallanes, Bravo-Barriga, Monteys, Martínez-de la Puente & Figuerola, 2024, suspected of feeding on birds based on its morphological traits (such as the third segment of the maxillary palp and the distribution and number of sensilla on the antenna), was one of the most commonly captured species in a recent study conducted in Spain using this method [72]. Moreover, studies investigating the role of *Culicoides* as avian malaria vectors have employed CDC traps without light but baited with live birds. These traps were placed near cages containing chickens (*Gallus gallus domesticus* Linnaeus, 1758), Japanese quails (*Coturnix japonica* Temminck & Schlegel, 1848), and zebra finches (*Taeniopygia guttata* Vieillot, 1817) [61, 71]. Other studies have placed traps in the canopy and near raptors' nests to investigate *Culicoides* species diversity and parasite prevalence at different heights [52, 73].

## Avian *Culicoides*-borne parasites

### *Haemoproteus*

*Haemoproteus* parasites are the most diverse group within the order Haemosporida. They consist of two subgenera: *Haemoproteus* (transmitted by Hippoboscidae flies) and *Parahaemoproteus* (transmitted by the biting midges of genus *Culicoides*) [29, 74]. Closely related to *Plasmodium*, *Haemoproteus* is a widespread blood protist often causing severe diseases, pathology, and even mortality in avian hosts, particularly in non-adapted species [29, 75, 76].

The life cycle of *Haemoproteus* (*Parahaemoproteus*) begins when an infected *Culicoides* injects the sporozoites into a susceptible avian host during blood feeding. These sporozoites invade tissue cells, initiating merogonic development. The first generation of meronts is typically found in the lungs, liver, spleen, gizzard, and skeletal muscle [29, 77–82]. The resulting merozoites may either invade other tissues, developing into megalomeronts, or enter erythrocytes, where gametocytes can be observed in blood smears, allowing for parasitaemia detection [29].

When a *Culicoides* bites an infected bird, it ingests mature gametocytes, which immediately undergo exflagellation in the insect’s midgut. Fertilization occurs rapidly, forming motile ookinetes. These ookinetes appear in the midgut within 1 h post-blood meal in species such as *Haemoproteus minutus* Valkiūnas & Iezhova, 1992*,* and other pale-staining *Haemoproteus* species, though they may still be seen 48 h after the blood meal for *Haemoproteus tartakovskyi* Valkiūnas, 1986 [49, 50, 83]. The ookinetes invade the midgut wall, developing into oocysts that become visible 3–7 days post-blood meal [41, 44, 49, 50, 83]. When mature, sporozoites are released and penetrate the hemocoel to reach the salivary glands [29]. This process is typically completed within 6–12 days post-infection [41, 44, 49].

Despite the described diversity of *Haemoproteus* parasites in birds, studies on vector competence remain limited. Currently, there are almost 180 described species of *Haemoproteus* [74, 77, 84, 85] and over 2000 genetic lineages according to the MalAvi database (accessed on 2025-04-08) [86]. Extensive research has focused on the genetic diversity, prevalence, and community composition of *Haemoproteus* across avian populations in different regions of the world [87–89].

Among studies that examined field-caught *Culicoides* combining xenomonitoring and microscopical examination of salivary gland preparation, natural vectors have been identified for only 11 *Haemoproteus* species (6.1% of those described) and 14 genetic lineages (0.7%). Confirmed natural vectors of *Haemoproteus* include *C. festivipennis*, *C. kibunensis*, *C. pictipennis*, *Culicoides reconditus* Campbell & Pelham-Clinton, 1960, and *C. segnis* [24, 25, 30–32, 57, 90]. Additionally, several species have tested positive for *Haemoproteus* DNA in field-caught parous females, including *Culicoides paolae* Boorman, 1996*, Culicoides scoticus* Downes & Kettle, 1952, *Culicoides alazanicus* Dzhafarov, 1961, *C. circumscriptus*, *Culicoides punctatus* Meigen, 1804, *Culicoides impunctatus* Goetghebuer, 1920, *Culicoides obsoletus* Meigen, 1818, and *Culicoides pallidicornis* Kieffer, 1919 [8, 25, 62, 69] (Table 1).

**Table 1:** *Haemoproteus* lineages and morphospecies molecularly identified in field-caught *Culicoides.*

*Haemoproteus* species (lineage)	*C. alazanicus*	*C. paolae*	*C. circumscriptus*	*C. festivipennis*	*C. impunctatus*	*C. kibunensis*	*C. obsoletus*	*C. pallidicornis*	*C. pictipennis*	*C. punctatus*	*C. reconditus*	*C. scoticus*	*C. segnis*	Country	Reference
*Haemoproteus* sp. (AEFUN03)			X											SP	[55]
*Haemoproteus* sp. (BLUTI09)			X											SP	[60]
*Haemoproteus* sp. (BRAM1)						X							X	RU	[90]
*Haemoproteus* sp. (CCF2)						X								LT	[24]
***H. fringillae* (CCF3)**													**X**	**LT**	[25]
*Haemoproteus* sp. (CCF4)											X		X	LT	[30]
*H. majoris* (CCF5)						X							X	LT	[24, 30, 31]
*H. noctuae* (CIRCUM01)	X		X	X										BG, SP	[58, 60]
*Haemoproteus* sp. (CIRCUM02)			X											SP	[60]
*Haemoproteus* sp. (CIRCUM03)	X		X											BG, SP	[58, 60]
*Haemoproteus* sp. (CIRCUM05)			X										X	LT, SP	[24, 60]
*H. pallidus* (COLL2)													X	LT	[32]
***H. homominutus* (CUKI1)**						**X**							X	CZ, **LT**	[24, 71]
*Haemoproteus* sp. (CULCIR1)			X											BG	[59]
*H. syrnii* (CULKIB01)						X			X					LT	[24, 25]
*Haemoproteus* sp. (CULKIB02)						X								LT	[25]
*Haemoproteus* sp. (CULKIB04)						X								LT	[24]
*Haemoproteus* sp. (CULKIB05)						X								LT	[24]
*Haemoproteus* sp. (CULPIC01)									X					LT	[24]
***Haemoproteus* sp. (CULPIC02)**									**X**					**LT**	[24]
*Haemoproteus* sp. (CUPAO-01)		X												SP	[55]
*H. majoris* (CWT4)							X						X	LT	[24, 32]
*H. hirundinis* (DELURB01)	X													BG	[58]
*Haemoproteus* sp. (GAGLA03)			X											SP	[55, 60]
*Haemoproteus* sp. (GAGLA05)			X											SP	[55]
***H. tartakovksyi* (HAWF1)**				X			X			X		X	**X**	**LT**, SK	[30, 31, 57, 62]
*H. concavocentralis* (HAWF2)			X											BG	[59]
*Haemoproteus* sp. (HAWF6)						X								LT, RU	[24, 90]
***H. belopolskyi* (HIICT1)**				**X**	X	**X**	X	X	X				X	**LT**, RU	[24, 25, 30, 90]
*H. balmorali* (LULU1)												X		LT	[57]
*Haemoproteus* sp. (ORORI01)	X													BG	[58]
*Haemoproteus* sp. (ORORI02)	X													BG	[58]
*H. majoris* (PARUS1)						X	X		X	X			X	LT	[24, 30, 31]
***H. pallidus* (PFC1)**						**X**				X				**LT**	[57]
*H. majoris* (PHYBOR04)						X								LT	[31]
***H. majoris* (PHSIB1)**													**X**	**LT**	[25, 30]
*H. lanii* (RB1)						X	X							LT, RU	[32, 57]
*H. attenuatus* (ROBIN1)				X			X							LT	[57]
***H. magnus* (ROFI1)**											**X**		X	CZ, **LT**	[24, 71]
*H. balmorali* (SFC1)	X													BG	[58]
*H. pallidus* (SFC3)	X													BG	[58]
*Haemoproteus* sp. (SFC4)							X							LT	[57]
***H. parabelopolskyi* (SYAT01)**						**X**			**X**			X	X	**DE, LT**	[7, 24, 32, 57]
***H. parabelopolskyi* (SYAT02)**				X		**X**			**X**			X		**DE, LT, RU**	[7, 24, 31, 90]
*H. pallidus* (SYAT03)						X			X					DE	[7]
***Haemoproteus* sp. (SYAT13)**						**X**			**X**					**LT**	[24]
***H. homogeneae* (SYAT16)**									**X**					**LT**	[24]
*Haemoproteus* sp. (SYAT35)						X			X					DE	[7]
*H. tartakovksyi* (SISKIN1)					X	X	X					X	X	LT, RU	[30, 32, 57, 126]
*H. syrnii* (STAL2)										X				LT	[30]
*H. minutus* (TUCHR01)						X								LT	[25]
***H. asymmetricus* (TUPHI01)**	X			**X**		**X**	X		**X**	X			**X**	BG, CZ, **LT**, SK, RU	[24, 25, 30–32, 57, 58, 62, 71, 90]
***H. minutus*** **(TURDUS2)**		X	X	X	X	**X**	X		**X**	X		X	**X**	SP, CZ, **LT**, BG, RU	[25, 30, 32, 55, 57, 59, 71, 90, 126]
*Haemoproteus* sp. (TURDUS3)													X	LT	[32]
*H. palloris* (WW1)						X		X					X	LT	[30, 31]
*H. majoris* (WW2)	X					X	X			X				BG, LT	[25, 31, 57, 58]

*BG* Bulgaria, *CZ* Czech Republic, *DE* Germany, *LT* Lithuania, *RU* Russia, *SP* Spain. *Culicoides* species were *Haemoproteus *sporozoites were reported are marked in bold. Records are restricted to lineages amplifying the 478-bp barcoding region of the parasite according to Hellgren et al., 2004 [108]

However, the number of confirmed *Haemoproteus* vectors (those with sporozoites identified in salivary gland preparations) remains limited. These species represent approximately 6% of the 117 *Culicoides* species found in Europe (based on the Fauna Europea dataset, updated by the world catalogue and recent species descriptions—Supplementary Table 1) [1, 91, 92].

Notably, *C. impunctatus* and *Culicoides nubeculosus* Meigen, 1830, have long been used in experimental infections to follow *Haemoproteus* development. This is mainly because *C. impunctatus* are usually found in high densities in nature, facilitating direct-exposure experiments, while *C. nubeculosus* is one of the few *Culicoides* species that were colonized, which also facilitates experiments. They have been proven competent vectors for several species [29, 41, 43, 44, 46, 48–50, 83, 93], although their role in natural transmission cycles remain a subject of ongoing debate.

### *Trypanosoma*

The genus *Trypanosoma* is dixenous; in other words, they alternate between vertebrate and invertebrate hosts, including *Culicoides*, during their life cycle [94]. For the *Trypanosoma* parasites that develop in *Culicoides*, the life cycle takes place on the insect gut, when the parasite multiplies, either as a free-floating stage or attached to the intestinal cell. In vertebrate hosts, *Trypanosoma* species persist extracellularly in the blood and lymphatic system [95].

During their life cycle, trypanosomes undergo distinct morphological transformations depending on the host and stage of development. Morphotypes are generally classified according to cell shape, nucleus-to-kinetoplast positioning, flagellum placement, and attachment to the cell [96, 97]. In vertebrate hosts, *Trypanosoma* usually occur as a trypomastigote, with epimastigote or amastigote stages occurring less frequently. In invertebrates, trypomastigote or epimastigote forms predominate, while promastigote and amastigote stages are rare [94].

Currently, 16 *Trypanosoma* subgenera are recognized. Avian trypanosomes are considered paraphyletic and are distributed among three subgenera: *Avitrypanum*, *Trypanomorpha*, and *Ornithotrypanum*, all of which are closely related to the mammalian subgenus *Megatrypanum* [94, 98]. At present, molecular data are available for 11 trypanosome named species that develop in avian hosts, categorized based on the size of their haematozoic trypomastigotes. The parasites with small haematozoic trypomastigotes, namely, *Trypanosoma anguiformis* Valkiūnas, Iezhova, Carlson & Sehgal, 2011, *Trypanosoma bennetti* Valkiūnas, Iezhova, Carlson & Sehgal, 2011, *Trypanosoma naviformis* Sehgal, Iezhova, Marzec & Valkiūnas, 2015, *Trypanosoma polygranularis* Valkiūnas, Iezhova, Carlson & Sehgal, 2011, and *Trypanosoma everetti* Molyneux, 1973; and the ones with large haematozoic trypomastigotes, namely *Trypanosoma avium* Votýpka, Szabová, Rádrová, Zídková & Svobodová, 2012, *Trypanosoma corvi* Stephens & Christophers, 1908 emend. Baker, 1976, *Trypanosoma culicavium* Votýpka, Szabová, Rádrová, Zídková & Svobodová, 2011, *Trypanosoma gallinarum* Bruce, Hamerton, Bateman, Mackie & Bruce, 1911, *Trypanosoma tertium* Fialová, Kapustová, Čepička & Svobodová, 2025, and *Trypanosoma thomasbancrofti* Slapeta, Morin-Adeline, Thompson, McDonnel, Sheils, Gilchrist, Votýpka & Vogelnest, 2016 [40, 94, 99–101].

Of these, only three species, *T. bennetti*, *T. everetti*, and *T. avium*, have been detected in *Culicoides* midges [22, 28, 52, 102]. Experimental evidence confirms that *C. nubeculosus* and *C. impunctatus* can serve as competent vectors of avian trypanosomes [22, 28, 102]. Moreover, trypanosomes are often found in field-caught biting midges, further supporting their role in parasite transmission [22, 28, 52] (Table 2).

## Molecular xenomonitoring of avian parasites in* Culicoides*

Molecular xenomonitoring of parasites in field-caught biting midges has become a valuable tool in identifying potential *Culicoides* vector species for avian blood parasites. This method involves polymerase chain reaction (PCR) amplification and sequencing of a fragment of the parasite DNA from field-caught midges, enabling detection without requiring the visualization of sporozoites or other developmental stages.

Typically, *Culicoides* females with a burgundy-coloured abdomen (parous and/or gravid) are prioritized for screening. This pigmentation generally indicates completion of at least one gonotrophic cycle, implying that the midge has already taken a blood meal [103], thus increasing the likelihood of harbouring parasites [29]. Nevertheless, species-specific reproductive strategies can complicate the visual identification of parous females. For example, newly emerged nulliparous females of *Culicoides imicola* Kieffer, 1913, may already exhibit abdominal pigmentation, potentially leading to misidentification of physiological status by external morphological characteristics [104]. Similarly, autogenous species such as *C. impunctatus* can produce their first batch of eggs without a blood meal [105, 106], bringing further challenges in distinguishing truly blood-fed individuals on pigmentation alone.

### Studies on *Haemoproteus*

Molecular screening of *Haemoproteus* parasites in parous *Culicoides* females has been conducted in multiple species, including *C. alazanicus*, *C. circumscriptus*, *C. kibunensis*, *C. festivipennis*, *C. pictipennis*, *C. obsoletus*, *C. scoticus*, *C. segnis*, *C. reconditus*, *C. punctatus*, *C. impunctatus*, *C. paolae*, *Culicoides deltus* Edwards, 1939, *C. pallidicornis*, *Culicoides fagineus* Edwards, 1939, *Culicoides albicans* Winnertz, 1852, *Culicoides fascipennis* Staeger, 1839*, Culicoides newsteadi* Austen, 1921*, Culicoides puncticollis* Becker, 1903, *Culicoides riethi* Kieffer, 1914, *Culicoides griseidorsum* Kieffer, 1918, and *Culicoides caucoliberensis* Callot, Krémer, Rioux & Descous, 1967 [24, 25, 30–32, 55, 57–60, 62, 70, 71]. Overall, at least 56 *Haemoproteus* lineages have been detected in *Culicoides*, identified through the amplification of the 478-base pair (bp) barcoding region of the mitochondrial cytochrome *b* gene [86, 107, 108] (Table 1). These studies have been mainly focused on European midge species, while data from other continents remain scarce [109–111]. Given the high degree of host specificity shown by *Haemoproteus* parasites, often restricted to particular bird families or even individual avian species [29], xenomonitoring in *Culicoides* could also provide indirect insights into vertebrate hosts on which these insects feed [5, 6, 25]. For example, *C. kibunensis* has been frequently found to be PCR-positive for *Haemoproteus* lineages primarily associated with birds of the Turdidae family, such as *H. minutus* TURDUS2 and *Haemoproteus asymmetricus* Valkiūnas, Ilgūnas, Bukauskaitė, Duc & Iezhova, 2021 TUPHI01 [24, 25], a finding that was further supported by host blood meal analysis of engorged females collected at the same study sites [5]. This approach demonstrates the potential of xenomonitoring not only for vector identification but also as a non-invasive method for monitoring avian biodiversity, especially in remote or protected areas where direct bird sampling may be restricted or unfeasible due to permitting constraints.

**Table 2:** *Trypanosoma* parasites molecularly identified in field-caught *Culicoides.*

*Trypanosoma* species (strain)	GenBank accession no.	*C. alazanicus*	*C. clastrieri*	*C. duddingstoni*	*C. festivipennis*	*C. impunctatus*	*C. kibunensis*	*C. nubeculosus*	*C. obsoletus*	*C. pallidicornis*	*C. pictipennis*	*C. reconditus*	*C. segnis*	Country	Reference	Origin
*Trypanosoma* sp. (Calaz187)	KY441578	X												CZ	[28]	FC
*Trypanosoma* sp. (Cpict335)	KY441579										X			CZ	[28]	FC
*Trypanosoma* sp. (Cclas340)	KY441580		X											CZ	[28]	FC
*Trypanosoma* sp. (Cfest115)	KY441577				X									CZ	[28]	FC
*Trypanosoma* sp.	MT236319										X			LT	[22]	FC
*Trypanosoma* sp.	MT236320								X					LT	[22]	FC
*Trypanosoma* sp.	MT236321								X					LT	[22]	FC
*Trypanosoma* sp.	MT236322										X			LT	[22]	FC
*Trypanosoma* sp.	MT236323						X		X		X			LT	[22]	FC
*Trypanosoma* sp.	MT236324										X			LT	[22]	FC
*Trypanosoma* sp.	MT236325											X		LT	[22]	FC
*Trypanosoma* sp.	MT236326						X							LT	[22]	FC
*Trypanosoma* sp.	MT236327						X							LT	[22]	FC
*T. avium*	MT269500												X	LT	[22]	FC
*T. avium*	PV018690										X		X	LT	[113]	FC
*T. theileri* group	PV018710										X			LT	[113]	FC
*T. theileri* group	PV018711										X			LT	[113]	FC
*T. culicavium*	PV018713				X									LT	[113]	FC
*T. bennetti* group	PV033375				X									LT	[113]	FC
*T. bennetti* group	PV033376			X	X		X							LT	[113]	FC
*T. bennetti* group	PV033377			X		X	X			X				LT	[113]	FC
*T. bennetti* group	PV033378			X										LT	[113]	FC
*T. bennetti* group	PV033379				X									LT	[113]	FC
*T. bennetti* group	PV033380								X					LT	[113]	FC
*T. bennetti* group	PV033381				X									LT	[113]	FC
*T. bennetti* group	PV033382			X										LT	[113]	FC
*T. bennetti* group	-						X				X		X	LT	[52]	FC
*T. avium*	-										X		X	LT	[52]	FC
*T. bennetti* (AAQU/SK/2000/APO7)	-							X						SK*	[28]	EX
*T. bennetti* group (AEMB/CZ/2002/PAS23)	-							X						CZ	[28]	EX
*T. avium* (ABUT/CZ/1999/BUT15)	-							X						CZ	[28]	EX
*T. everetti*	MT236328					X								LT	[22]	EX
*T. everetti*	MT236329							X						LT	[22]	EX
*T. everetti*	MT236330							X						LT	[22]	EX
*T. everetti*	MT236331							X						LT	[22]	EX

*CZ* Czech Republic, *LT* Lithuania, *SK* Slovakia. * Strain of origin. FC: field-caught. EX: experiment infection

In addition to host–parasite interactions already known, xenomonitoring can reveal novel or unexpected associations when the origin of a *Culicoides* blood meal is identified and the same insect individual harbours parasite lineages not previously recorded in avian hosts. For example, it was demonstrated that *Culicoides* individuals that had fed on long-eared owls (*Asio otus* Linnaeus, 1758) harboured the *Haemoproteus noctuae* Celli & Sanfelice, 1891 lineage CIRCUM01. Likewise, midges carrying the *Haemoproteus *sp. CIRCUM03 lineage were shown to have recently fed on Eurasian magpies (*Pica pica* Linnaeus, 1758) [58]. The initial suggestion that CIRCUM01 is specific to long-eared owls was later confirmed when the lineage was molecularly identified in this avian species [43].

### Studies on *Trypanosoma*

Molecular identification of trypanosomes in *Culicoides* biting midges is commonly conducted using a nested PCR protocol that amplifies a DNA fragment encoding the SSU *18S* rRNA [100, 112]. This approach has enabled researchers to confirm the role of *Culicoides* as vectors of avian trypanosomes, particularly those within the *T. bennetti*/*everetti* group.

Studying trypanosomatids in *Culicoides* captured in the forest canopy nearby nests of raptor birds using PCR-based detection, at least eight *Culicoides* species (*Culicoides duddingstoni* Kettle & Lawson, *C. impunctatus*, *C. obsoletus* group, *C. pallidicornis*/*subfasciipennis*, *C. festivipennis*, *C. kibunensis*, *C. pictipennis*, and *C. segnis*) were detected to harbour DNA of the *T. bennetti*/*everetti* group; *T. avium* was detected in *C. pictipennis* and *C. segnis*; and *T. culicavium* in *C. festivipennis* [52, 113]. Similarly, a study in Czechia detected *Trypanosoma* DNA in four out of 1184 trapped biting midges. One sequence, *Trypanosoma* sp. Calaz187 (from *C. alazanicus*, GenBank KY441578), was found to be identical to other sequences from the lineage VIII, previously isolated from avian hosts. Additionally, sequences of *Trypanosoma* sp. Cpict335 and *Trypanosoma* sp. Cclas340 (from *C. pictipennis* and *Culicoides clastrieri* Callot, Kremer & Deduit, 1962, GenBank KY441579, KY441580) were identical and clustered within the lineage VI, while *Trypanosoma* sp. Cfest115 (from *C. festivipennis*, GenBank KY441577) formed a branch closely related to lineage VI [28, 40].

It is noteworthy that not all detected trypanosomatids in *Culicoides* are dixenous. Approximately one-third of PCR-positive samples correspond to monoxenous genera, such as *Obscuromonas*, *Sergeia*, *Herpetomonas*, and others, which are restricted to a single insect host and not usually transmissible to vertebrates [114].

Different ecological factors appear to influence the prevalence of *Trypanosoma* parasites in biting midges. For example, the prevalence rates in field-caught females varies between 6.8% at the ground level [22] and 24% in canopy samples collected near raptor nests [52]. In addition, the abundance of flying haematophagous insects captured in avian nests, including *Culicoides* and blackflies, has been positively correlated with the prevalence of *Trypanosoma* in blue tit nestlings [33].

### Limitations of molecular xenomonitoring studies

Although molecular xenomonitoring has proven valuable for detecting parasite DNA in biting midges, this method alone does not allow the confirmation of vector competence. Detection of parasite DNA may result from residual genetic material of abortive forms that persist in the insect for a long time after feeding on an infected host [115]. For example, *Plasmodium* and *Leucocytozoon* DNA were detected in *Culicoides* [24, 25, 58, 62, 69, 110, 116, 117], despite these parasites being transmitted by other vector groups: mosquitoes and black flies, respectively (except for *Leucocytozoon caulleryi* Mathis & Léger, 1909, which is transmitted by *Culicoides* and not found in Europe). This highlights the need for additional validation when assessing vector status.

To confirm a *Culicoides* species as a competent vector, it is crucial to detect the infective stages of parasites: *Haemoproteus* sporozoites in the salivary glands and *Trypanosoma* metacyclic forms in the gut of *Culicoides*. These stages are acquired by the vector after feeding on an infected bird; they are responsible for initiating infection in the vertebrate hosts and are critical indicators of successful parasite development and transmission potential.

## Studies identifying competent *Culicoides* vectors

### Methodologies for detecting infective parasite stages

While time-consuming, dissections of field-caught *Culicoides* females with burgundy abdominal pigmentation (indicative of parity and at least one blood meal—for most species, see discussion above) remain the gold standard for demonstrating the development and transmission of avian *Haemoproteus* [29] and *Trypanosoma* [22] within a vector*.* Currently, investigations focusing on *Haemoproteus* parasites are primarily limited to Lithuania and the Kaliningrad Oblast, Russia [24, 25, 30–32, 57, 90], while research on avian trypanosomes is largely restricted to Czechia [28]. This results in a limited understanding of the *Culicoides* species involved in avian parasites transmission across Europe. This classical parasitological technique requires specialized training and expertise, especially for dissecting tiny insects like *Culicoides*.

Dissections of field-caught *Culicoides* can be combined with molecular xenomonitoring to enhance detection efficiency. In this integrative approach, females exhibiting abdominal pigmentation are dissected, and their thoraxes (containing salivary glands) are gently smeared in a drop of 0.9% saline solution, air-dried, fixed with absolute methanol, and stained using 4% Giemsa for 1 h [22, 29, 42]. These smears are examined microscopically for *Haemoproteus* sporozoites, while the remaining insect tissues can be processed via PCR to confirm parasite identity. This dual strategy reduces microscopic workload by limiting detailed analysis to PCR-positive individuals. The detection of sporozoites in thorax smears confirms the ability of the *Culicoides* species to support sporogonic development, thereby indicating its competence as a vector of *Haemoproteus*.

Similarly, the presence of *Trypanosoma* metacyclic trypomastigotes in the gut serves as evidence of vector competence. Dissection protocols for *Trypanosoma* are broadly similar to those used for detecting haemosporidian ookinetes. The midgut and hindgut should be extracted from the abdomen and gently crushed in a saline solution for further microscopic examination on Giemsa-stained preparations [22]. Unlike *Haemoproteus* ookinetes, which are typically observed in the initial stages of infection (up to 2–4 days post-infection), vector-specific *Trypanosoma* infections are detected later, after defecation has occurred (up 2–9 days post-infection).

A key advantage of this approach in kinetoplastid research is the ability to establish parasite cultures from dissected insects. These cultures can be cryopreserved and maintained for further study. A range of culture media are available for establishing new kinetoplastid isolates from insect guts [53, 118], and similar methods may also be applied to isolate trypanosomes directly from avian hosts.

### Main results obtained using dissections of field-caught *Culicoides*

In addition to confirming infection, microscopical examination of salivary gland preparations enables the morphological characterization of *Haemoproteus* sporozoites. Even though this parasite stage exhibits limited distinguishing features for species-level identification, differences in size and shape may indicate their taxonomic grouping [24, 43]. It has been hypothesized that larger sporozoites are associated with pale-staining *Haemoproteus* species (e.g., *H. minutus*, *Haemoproteus homominutus* Valkiūnas, Ilgūnas, Bukauskaitė, Chagas, Bernotienė, Himmel, Harl, Weissenböck & Iezhova, 2019, and *H. asymmetricus*), while smaller and thinner sporozoites may belong to species such as *Haemoproteus belopolskyi* Valkiūnas, 1989, *Haemoproteus parabelopolskyi* Valkiūnas, Križanauskienėm Iezhova, Hellgren & Bensch, 2007, and *Haemoproteus homogeneae* Valkiūnas, Ilgūnas, Bukauskaitė, Chagas, Bernotienė, Himmel, Harl, Weissenböck & Iezhova, 2019 [24]. Further research is needed to validate this morphological hypothesis and enhance the understanding of *Haemoproteus* taxonomy across developmental stages.

Molecular research on *Trypanosoma* parasites in *Culicoides* has indicated that prevalence of trypanosomatids in field-caught biting midges can reach 11.1% [52]. However, studies incorporating microscopical examination of gut preparations in field-caught insects remains scarce, with most relying exclusively on molecular detection [22, 28, 52].

The commonly used *18S* rRNA gene lacks the resolution to distinguish between all avian *Trypanosoma* subgenera (e.g., *Avitrypanum*, *Trypanomorpha*, *Ornithotrypanum*) and often fails to differentiate closely related species such as *T. avium* versus *T. thomasbancrofti* or *T. bennetti* versus *T. everetti*, due to unresolved polytomies in phylogenetic trees [22, 40, 119–121]. For deeper phylogenetic resolution, alternative genes or phylogenomic approaches are recommended.

Throughout their development in insect hosts, *Trypanosoma* exhibit morphological variability [97]. Although, the fully grown haematozoic trypomastigotes tend to exhibit conserved features, including body shape and size, and the morphology and position of key organelles such as the kinetoplast, flagellum, and undulating membrane [97]. Consequently, microscopic examination remains a valuable diagnostic tool, especially when parasite loads are low and molecular methods may fail to detect parasite DNA [112]. The combination of microscopic and molecular techniques can be considered as an ideal approach for studying *Trypanosoma* in *Culicoides* vectors.

### Experimental infections of *Culicoides*

Experimental infection studies offer essential insights into vector competence and allow for a detailed understanding of the parasite’s life cycle within their insect vectors. While several *Culicoides* species have been used in experimental infections with *Haemoproteus* parasites [27, 41, 93], most of these studies were historically conducted mainly in North America during the twentieth century [93]. More recently, experimental work has focused on populations in Lithuania and Kaliningrad Oblast, Russia [41–46, 48–50]. Similarly, experimental studies of *Trypanosoma* transmission by *Culicoides* in Europe have been limited to a few geographical locations and two species of biting midges: *C. nubeculosus* and *C. impunctatus* [22, 28].

In Europe, experimental studies have predominantly utilized wild *C. impunctatus* and laboratory-reared *C. nubeculosus*, both of which have been shown to support sporogonic development of at least 19 *Haemoproteus* species (C.* impunctatus*: *H. minutus*, *H. noctuae*, *H. belopolskyi*, *Haemoproteus balmorali* Peirce, 1984, *Haemoproteus majoris* Laveran, 1902*, Haemoproteus motacillae* Bennett & Bishop, 1990*, Haemoproteus pallidus* Valkiūnas & Iezhova, 1991,* Haemoproteus nucleocondensus* Križanauskienė, Iezhova, Palinauskas, Chernetsov & Valkiūnas, 2012*, H. tartakovskyi*, *Haemoproteus dolniki* Valkiūnas & Iezhova, 1992, *Haemoproteus fringillae* Labbé 1894, *Haemoproteus lanii* Mello, 1936; *Culicoides nubeculosus*: *H. noctuae*, *Haemoproteus syrnii* Mayer, 1910*, H. tartakovskyi*, *Haemoproteus handai* Maqsood, 1943, *Haemoproteus attenuatus* Valkiūnas, 1989, *H. minutus*, *H. motacillae*, *Haemoproteus pastoris* Mello, 1935, *Haemoproteus homopalloris* Chagas, Bukauskaitė, Ilgūnas, Iezhova & Valkiūnas, 2018, *H. belopolskyi*, *Haemoproteus hirundinis* Sergent & Sergent, 1905, *H. nucleocondensus*, *H. lani*, *H. majoris*) [39, 41, 42, 44–46, 83, 122]. Additionally, both species have been shown to support the metacyclic development of trypanosomes from the *T. bennetti*/*everetti* group [22, 28, 29, 39, 41–46, 48–50, 63, 83, 122].

Experimental infections typically begin with selecting an appropriate avian donor, ideally a bird displaying mature *Haemoproteus* gametocytes in blood smears and with gametocytaemia of 0.1–0.5% [41, 44] or a bloodstream infection with *Trypanosoma* [22]. These donors can be directly exposed to the biting midges [22, 46], or *Culicoides* can fed through a membrane on blood with cultured parasites, for experiments with trypanosomatids [28]. When birds with higher *Haemoproteus* gametocytaemia are used, *Culicoides* experience elevated mortality, indicating that these parasites are pathogenic to the vector [38, 39]. However, there is currently no data on how *Trypanosoma* parasitemia affects vector survival. Importantly, it remains unknown whether *Haemoproteus* and *Trypanosoma* parasitaemia influences the feeding preference of biting midges, as no experimental tests have been conducted to address this host–parasite interaction.

Experimental infections have been conducted using both field-caught and laboratory-reared *Culicoides*. For field-based studies, birds are typically gently restrained in gloved hands by researchers and exposed to feeding midges under natural or semi-controlled conditions [29]. While these studies confirm the vector competence of wild *C. impunctatus,* interpretation of these findings should be cautious. Under natural conditions, *C. impunctatus* may not play a major role in the transmission of avian *Haemoproteus* and *Trypanosoma* parasites due to its mammalophilic feeding preference [105, 123]. Similar limitations apply to studies based on laboratory colonies of *C. nubeculosus*.

## Concluding remarks and future directions

The identification of wildlife parasite vectors remains an increasingly prominent research topic within the broader field of host–vector–parasite interactions and disease transmission ecology. Despite recent advancements, substantial knowledge gaps persist, particularly regarding the vector ecology of *Culicoides* biting midges. One critical area for further research concerns their blood-feeding preferences. Identifying the host species or individuals they primarily target within ecological communities is essential to understanding transmission pathways. This question can be addressed by analysing blood-engorged females, combined with data on local avian community composition and abundance. Additionally, the detection of haemosporidian and trypanosome parasites in engorged midges can reveal blood-feeding patterns due to the host specificity of many of these parasites, especially for *Haemoproteus* parasites [124]. Understanding host preferences can also contribute to the identification of *Culicoides* species capable of acting as bridge vectors of zoonotic pathogens [6, 125].

Beyond host–*Culicoides* association, further research is needed to determine the degree of specificity between individual parasite lineages and specific *Culicoides* taxa, as well as to assess the effects of such infections on the biology and fitness of the insect host. Investigating the impact of host infection status and parasitaemia load on the biology of *Culicoides* feeding on infected hosts may help to clarify how parasites influence key vector traits such as activity patterns, survival, fecundity, and biting rates. These studies are essential for gaining a deeper understanding of the ecological and epidemiological dynamics of parasite transmission in the natural environment.

To advance this field, future research should adopt integrative and multidisciplinary approaches. Investigations should include molecular xenomonitoring to identify parasite DNA in field-caught insects, dissection of field-caught parous *Culicoides* females to confirm infective stages, microscopical analysis of salivary glands and midgut preparation, and experimental infection to verify vector competence. There is also a pressing need to expand research efforts beyond the currently studied regions. Most available data derive from a limited number of geographical locations and epidemiological contexts where specialized research groups operate, often with access to the technical expertise required for these procedures. Expanding this work across a broader geographical scale is essential, particularly in the face of global climate change. Shifting ecological conditions are likely to reshape host, vector, and parasite communities, generating novel epidemiological scenarios that require proactive investigation.

Finally, ensuring the long-term sustainability of this research requires investment in capacity building to effectively transmit this knowledge to future generations. Future projects should prioritize capacity building, hands-on training, and knowledge transfer to support the next generation of scientists. Developing and disseminating technical skills in vector surveillance, parasite detection, and experimental manipulation will strengthen global research efforts and contribute to a more comprehensive understanding of parasite–vector interactions in wildlife systems.

## Supplementary Information

**Additional file 1: Table S1.** List of *Culicoides* species in Europe according to the Fauna Europea dataset (Jong 2016), updated regarding nomina dubia from the catalogue of the world fauna (Borkent and Dominiak 2020), the last update of the catalogue (Borkent, Dominiak, and Díaz 2022), and the new species described since 2016 (date of the Fauna Europea dataset).

## Data Availability

Data supporting the main conclusions of this study are included in the manuscript.
